# Investigation of age- and sex-related changes in cancellous bone density in the human spine using CT

**DOI:** 10.1371/journal.pone.0358344

**Published:** 2026-09-28

**Authors:** Guido Schröder, Aleksander J. Owczarek, Estelle Akl, Andreas Götz, Steffi S. I. Falk, Thomas Mittlmeier, Laura Hiepe, Andreas Büttner, Michael Dau, Daniel Wolter, Julian Ramin Andresen, Reimer Andresen, Hans-Christof Schober, Änne Glass

**Affiliations:** 1 Clinic for Orthopaedics and Trauma Surgery, Sana Hospital Bad Doberan, Academic Teaching Hospital of the University of Rostock, Hohenfelde, Germany; 2 Health Promotion and Obesity Management Unit, Department of Pathophysiology, Faculty of Medical Sciences in Katowice, Medical University of Silesia, Katowice, Poland; 3 Institute for Diagnostic and Interventional Radiology, Pediatric and Neuroradiology, Rostock University Medical Center, Rostock, Germany; 4 Institute for Biomedical Technology, Rostock University Medical Center, Warnemuende, Rostock, Germany; 5 Department of Trauma Surgery, Hand and Reconstructive Surgery, Rostock University Medical Center, Rostock, Germany; 6 Institute of Anatomy, Rostock University Medical Center, Rostock, Germany; 7 Institute of Legal Medicine, Rostock University Medical Center, Rostock, Germany; 8 Department of Oral & Maxillofacial Plastic Surgery, Rostock University Medical Center, Rostock, Germany; 9 Division of Trauma Surgery, Department of Orthopaedics and Trauma Surgery, Medical University of Vienna, Waehringer Guertel, Vienna, Austria; 10 Institute of Diagnostic and Interventional Radiology/Neuroradiology, Westkuestenklinikum Heide, Academic Teaching Hospital of the Universities of Kiel, Luebeck and Hamburg, Heide, Germany; 11 OrthoCoast, Medical Office of Orthopedics and Osteology, Wolgast, Germany; 12 Institute for Biostatistics and Informatics in Medicine and Ageing Research, Rostock University Medical Center, Rostock, Germany; Federal University of Minas Gerais: Universidade Federal de Minas Gerais, BRAZIL

## Abstract

Studies of cancellous bone density across all spinal segments in relation to age and sex remain limited. This investigation aimed to quantify vertebral cancellous bone density in Hounsfield units (HU), to explore age‑ and sex‑related bone loss, and to compare HU measurements with bone mineral density (BMD) and bone volume fraction (BV/TV). Cancellous bone density was assessed in 71 spines (CT) covering vertebrae C3-L5 and the age range from 4 to 102 years. HU values were derived from mid‑vertebral regions, and in addition, BMD (L1-L3) and BV/TV measurements (Th12-L2, micro‑CT, 50 spines) in broad subsets of samples. The association between bone density and age was investigated by HU profiles along the spine in age groups and by regression approaches. HU values decreased with age (p < 0.001) and from cranial to caudal levels (p < 0.001), for both sexes (p < 0.001), with a marked reduction at the thoracolumbar junction beyond 60 years. Regression and cluster analyses revealed two phases of HU density loss with ongoing age, the second occurring in women (older than 64 years), later than in men (after 56 years) (p < 0.001). HU values were correlated with BMD (r = 0.86; 95% CI: 0.72–0.94) and with BV/TV (r = 0.51; 95% CI: 0.17–0.75). Density loss of bone with age is significant for BMD (for both sexes, p < 0.001) and for BV/TV (p ≤ 0.021), too, with an earlier reduction detected in the microarchitecture. These findings highlight the need for sex‑specific assessment and prevention strategies in osteoporosis research and clinical care.

## 1 Introduction

Osteoporosis (OP) is globally one of the most common diseases in the elderly and one of the most significant causes of morbidity, reduced quality of life, and mortality in advanced age [1]. The SCOPE *study* [2*] reported that osteoporosis affects approximately 5.6% of people aged 50 years and older across the* EU*, the United Kingdom, and Switzerland, corresponding to 22% of women and 7% of men.* It is marked by a reduction of bone density and a deterioration of the microarchitecture of bone, which increases the risk of fractures, especially in the spine, the femoral neck and the distal radius [3]. Therefore, early identification of individuals at risk and precise quantification of bone loss are crucial for the prevention, diagnosis and treatment of the disease.

Traditionally, bone density is determined by dual X-ray absorptiometry (DXA). This method is regarded as the gold standard [4], but its value is limited by the superimposition of soft tissue and degenerative changes. Over the last few years, quantitative computed tomography (QCT) has been established as a complementary method [5] that permits three-dimensional registration of bone density and provides valuable information, especially for the analysis of trabecular structures. The American College of Radiology (ACR) has recommended diagnostic criteria for OP based on QCT of the lumbar spine (LS) [6]: normal > 120 mg/cm^3^; low bone mass 80–120 mg/cm^3^; and osteoporosis < 80 mg/cm^3^. Compared to DXA, QCT-based measurement of volumetric bone density in the LS of elderly patients and postmenopausal women yielded a higher detection rate for OP [7].

In clinical routine, Hounsfield units (HU), which can be extracted from standard CT images, are being used to an increasing extent as a surrogate parameter of bone density [8,9]. The measurement of HU on non-contrasted CT’s of the spine, a practical yet not as established a method as DXA, is regarded as a valid procedure for the estimation of the risk of osteoporosis [10,11]. Buenger et al. [12] investigated thoracolumbar vertebrae and described a correlation between bone density determined by QCT and HU. Schreiber et al. [13] noted a significant correlation between T-values of DXA measurements and HU in the same vertebral body. The advantage of HU-based analyses is that HU values can be obtained retrospectively from pre-existing CT datasets without additional radiation exposure or special investigation protocols [8]. Furthermore, HU measurements can be used as an effective diagnostic method instead of DXA scans when the clinician wishes to select a suitable mode of treatment for patients with spinal implants or degenerative spines [14].

Apart from bone density, the assessment of bone volume fraction (BV/TV) is gaining importance. Especially, high-resolution micro-CT procedures permit a detailed analysis of trabecular microstructure and create new perspectives for understanding age- and sex-specific bone loss [15–18].

This investigation was conducted to quantify cancellous bone density along the entire spine using HU measurements, and to compare these findings with QCT‑derived bone mineral density and micro‑CT bone volume fraction (BV/TV). We aimed to depict the course of age‑related bone loss by analyzing human spines from a broad age range (4–102 years), and to get in-depth knowledge about the sex-specificity of courses, too. Comprehensive analyses comparing HU, BMD, and BV/TV measurements obtained partly from the same vertebrae are still rare, leaving a gap in the direct comparability of morphological, radiological, and structural bone parameters. Thus, we merged data from three different samples to cover diverse ages and radiological imaging methods. The results are expected to deepen our understanding of vertebral bone physiology and to improve the diagnostic interpretation of HU values for clinical OP assessment.

## 2 Materials and methods

### 2.1 Study design and ethics

The present investigation was an observational, cross-sectional analysis, which combined clinical imaging with pre-clinical imaging of cadavers. In total, N = 71 spines (23 autopsied cadavers of young persons, 22 middle-aged trauma surgery patients, and 26 body donors) were analyzed. The data collection began on 20 March 2025, and the investigator had access to the radiological information of the individual participants during the data collection period. Joining three different samples for statistical analysis of image data allowed for covering a broad spectrum of age, even though excluding this way the chance of a conventional study design. The investigation was conducted at the University Medical Center in accordance with the Declaration of Helsinki and was approved by the institutional ethics committee (A2017‑0072). All procedures involving human tissue followed institutional and legal requirements for post-mortem research. Details of tissue extraction and preservation were based on established methods previously published by our group [15].

### 2.2 Inclusion and exclusion criteria

Spines showing no anatomic deformities or pathological bone conditions (e.g., tumors, metastases, Paget’s disease, or dysplastic growth disorders) were included. Samples containing foreign material from previous surgery or vertebral fusions were excluded. An interdisciplinary team of anatomists and senior physicians in orthopedics and trauma surgery applied the inclusion and exclusion criteria. Following macroscopic inspection, standardized computed tomography (CT) scans were obtained for all specimens. Two independent board-certified radiologists with extensive expertise in osteological imaging evaluated the CT images in a double‑blind manner to ensure objectivity.

### 2.3 Extraction of spines and cancellous tissue

After the post‑mortem interval, cadavers were perfused via the left femoral artery using 96% ethanol at 0.5 bar pressure, followed by immersion in 0.5% aqueous phenol solution. Spines were exposed and extracted in prone position and stored in 70% ethanol at 4 °C until further imaging and sampling. Cylindrical cancellous bone specimens were obtained from the central ventromedial region of vertebral bodies using a Jamshidi® needle (8 gauge, 3.26 mm diameter). CT examinations were used to verify the extraction site and to exclude compromised segments. All samples were stored in 1.5 ml Eppendorf tubes for subsequent micro‑CT analysis.

### 2.4 Diagnostic imaging

#### 2.4.1 CT and QCT.

In order to create realistic conditions for clinically accurate and anthropometric measurements, the extracted spines and segments of vertebrae were submerged carefully in a PLEXIGLAS® (PMMA) water phantom measuring 125 cm in length and 25 cm in diameter (Fig 1). Care was taken to ensure there were no air pockets. The donor spines were subjected to a high-resolution spiral CT investigation (GE Revolution EVO/64-slice CT/lateral scanogram) with an axial slice thickness of less than 1 mm. Axial, coronal, and sagittal reformations were obtained with a slice thickness of 2 mm. Quantitative assessment of trabecular bone density in each vertebral body (in Hounsfield units, HU) was performed using a manually positioned region of interest (ROI) within the trabecular compartment. The ROI was drawn as large as possible while carefully excluding the cortical shell to avoid partial volume effects. To obtain a representative measure of vertebral bone density, the mean attenuation value derived from the axial, coronal, and sagittal planes was used for further analysis. The resulting HU values reflect the combined attenuation of trabecular bone and the resident bone marrow, without dedicated segmentation or thresholding of red versus yellow marrow. Given that only two donors in our cohort were younger than 19 years, primary HU analyses were performed in subjects aged ≥19 years, in whom vertebral marrow predominantly follows the adult fatty pattern. The bone mineral density (BMD) of cancellous bone was determined on quantitative CT (GE Revolution EVO/64-slice computed tomography device and Mindways software (version 4.2.3) 3D Volumetric QCT Spine, Austin, TX, USA). Measurements were performed on a volume block at the levels of L1, L2, and L3. The mean value – given in mg/cm^3^ - was used to estimate the risk of osteoporosis.

**Fig 1:**
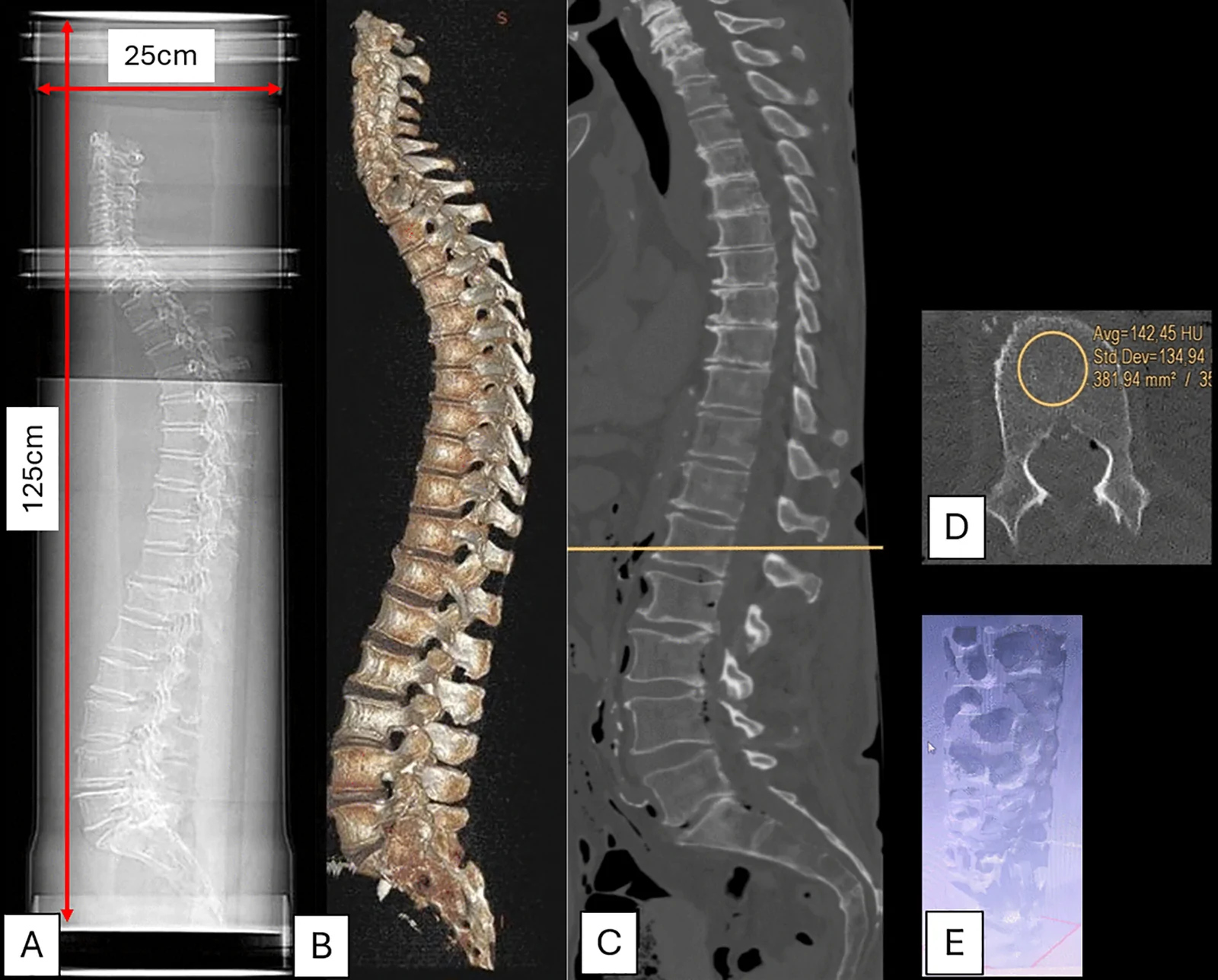
Visualization of the experimental setup, image reformation, and density measurement. Position of an embedded spine within the plexiglass-water phantom. (A) To simulate the soft-tissue envelope, the donor spines were embedded in water with as little air as possible; the phantom has a transverse diameter of 25 cm and a length of 125 cm. (B) The 3D CT reconstruction of the spines was used to detect pathologies. (C) CT of the spine of a trauma patient and exemplary measurement of Hounsfield units at the level of L1. (D) Density determination in HU was performed using an individually placed region of interest (ROI) positioned mid-vertebrally in each vertebral body of the vertebral column. L1 is shown as an example. (E) Micro-CT reconstruction of a mid-vertebral trabecular bone biopsy. L1 is shown as an example.

#### 2.4.2 Micro-CT images and evaluation of microarchitecture.

A total of 150 cancellous bone samples (from Th12 and L1–L2) were scanned using a high-resolution micro‑CT system (SkyScan 1172; RJL Micro & Analytic GmbH, Karlsdorf‑Neuthard, Germany). Flat‑field correction was applied, and reference phantoms (0.25 g/cm^3^ and 0.75 g/cm^3^) served for calibration. Acquisition settings were as follows: aluminum filter 0.5 mm, pixel size 19.9 µm, isotropic voxel size 35 µm, source 100 kV / 100 µA, field‑of‑view 70 mm. *A standardized* volume of interest (VOI*) was defined within the trabecular compartment to exclude the cortical shell.* Bone volume fraction (BV/TV, %) was calculated as the ratio of segmented bone volume to total tissue volume within the trabecular compartment and was quantified within this defined VOI. The 3D reconstruction of a trabecular bone biopsy core can be seen in (Fig 1).

### 2.5 Statistics

Cancellous bone density in HU and bone mineral density (BMD, mg/cm^3^) were obtained from CT images, and bone volume fraction (BV/TV, %) was measured on micro-CT. HU values were documented in 71 HU data records (for the vertebrae from C3 to L5; 34 female, 37 male), with 58 BMD values (mean L1-L3; 30 female, 28 male) available, and 50 BV/TV data records (Th12-L2; 23 female, 27 male). HU, BMD, and BV/TV values were analyzed with regard to age (years) and three more or less equal age groups in the context of osteoporosis (4−30, 31−60, 61−102 years). Due to skewed data distribution in some subgroups, descriptive statistics were reported as median with quartiles (lower – Q_1_, upper – Q_3_), sample size (n), as well as mean. Cervical HU values (C3-7) could be considered normally distributed, but thoracic and lumbar values could not. Statistical tests of mean differences of bone density between subgroups are non-parametric: for sex groups (Mann-Whitney U test), age groups (Kruskal-Wallis test followed by pairwise U tests, with Bonferroni-adjusted p values), and location in the spine (Friedman test, followed by pairwise Wilcoxon tests). To assess the relationship between HU, BMD, and BV/TV density measurements, Pearson’s correlation coefficient of individual means was given with the respective confidence interval (95% CI: lower-upper limit). To estimate the age-related change in HU values, a linear regression model was fitted to the aggregated (C3-L5) HU values, adjusted for sex; the same was done for BMD values. The adjusted regression coefficient b was tested for being zero. B was z-standardized (b^0,1^) for comparison between the radiological measurement procedures.

To find a cut-off age that divides “high HU values in young age” from “low HU values in advanced age,” we first performed k-means cluster analyses (based on the MacQueen algorithm) separately for women and men. HU values and age data were z-standardized and clustered with k = 3 start clusters. The two final clusters were used for the ROC curve analysis of age with 1,000 bootstrapping samples, maximizing Youden’s index to find an age-related cut-off separating higher and lower HU values. The resulting age (R package‚ *cutpointr*‘) is highlighted in the local (loess smooth) regression plot for each vertebra from C3 to L5 for both sexes. Plots show the curve of the best loess fit to the data as a regression approach, which is not restricted to a linear form. The curve represents the association between HU values and age with the corresponding confidence bands.

The data was analyzed with IBM SPSS Statistics (version 29.0), and with R for cluster, ROC curve, and local regression analyses. All statistical tests were two-sided and p-values < 0.05 were considered significant.

## 3 Results

In the present investigation, the density of cancellous bone in Hounsfield units (HU) was determined in persons aged 4–102 years to explore bone loss with age along the spine C3 to L5, and the influence of sex thereon. Results were compared with the widely reported cancellous bone mineral density (BMD, mg/cm^3^) as well as trabecular bone volume fraction (BV/TV, %). In all, 1562 vertebrae obtained from 71 spines (34 female, 37 male) with a median age of 59 years (Q_1_ = 36, Q_3_ = 78) and a BMI of 20.6 kg/m^2^ (17.7, 24.9) were investigated. The three included samples differed in terms of age (forensic medicine cadavers: 28 years (21, 37), patients: 58 years (50, 64), body donors: 80 years (78, 84); p < 0.001), but not in terms of BMI (p = 0.283). 39% of the 71 spines (19 female, 9 male) were osteoporotic (68% female vs. 32% male), with either a BMD < 80 mg/cm^3^ or, if BMD measurements were not available (n = 3), with converted HU values (mean L1-L3) into BMD values [12].

### 3.1 Different cancellous bone density profiles (HU) on different levels in the age groups

Cancellous bone density (HU) determined on CT reduces from the cranial to the caudal aspect (p < 0.001): The highest HU values were found in the CS = 266 HU (Q1 = 184, Q3 = 354), with a reduction noted in the TS = 198 HU (104, 300), and the lowest values registered in the LS = 159 HU (75, 259) (S1 Table). To assess the reduction with ongoing age, we depicted median HU profiles along the spine (C3-L5) for three age groups (4–30 years, 31–60 years, 61–102 years), n ≥ 15 each. In young individuals we noted median values ≥ 300 HU in the entire spine: C3 = 384 HU (337, 469); L5 = 328 (230, 358) (Fig 2A). The sample was quite balanced in sex (6 women, 9 men), and none of the spines was osteoporotic. In the middle age group (31–60 years) we registered overall medians > 200 HU: C3 = 375 HU (296, 426); L5 = 226 HU (151, 295) (Fig 2B). Cancellous bone density is not stabilized in the lower spine as in younger persons but continues to decline: the majority of thoracic and lumbar vertebrae were found to be different between the two younger age groups (p-values for Th4 0.011, Th5 0.028, Th7 0.039, Th10 0.026, Th11 0.045, Th12 0.014, L2 0.042, L4 0.013, L5 0.013), but not the cervical ones. The sample included more men (16 vs. 6), and one osteoporotic spine. Older individuals (> 60 years) had reduced cancellous bone densities: C3 = 215 HU (173, 267); L5 = 66.0 HU (53.5, 99.0), which were significant lower in all 22 vertebrae compared to younger subjects (all p < 0.001) (Fig 2C). The sample was female-dominated (22 vs. 12 men), and 79% (27/34) of the spines were osteoporotic. The OP reference value of 100 HU was first undercut in Th6. In this age group both, age and OP impact on bone density, so that observed HU level changes can’t be attributed exclusively to age.

**Fig 2:**
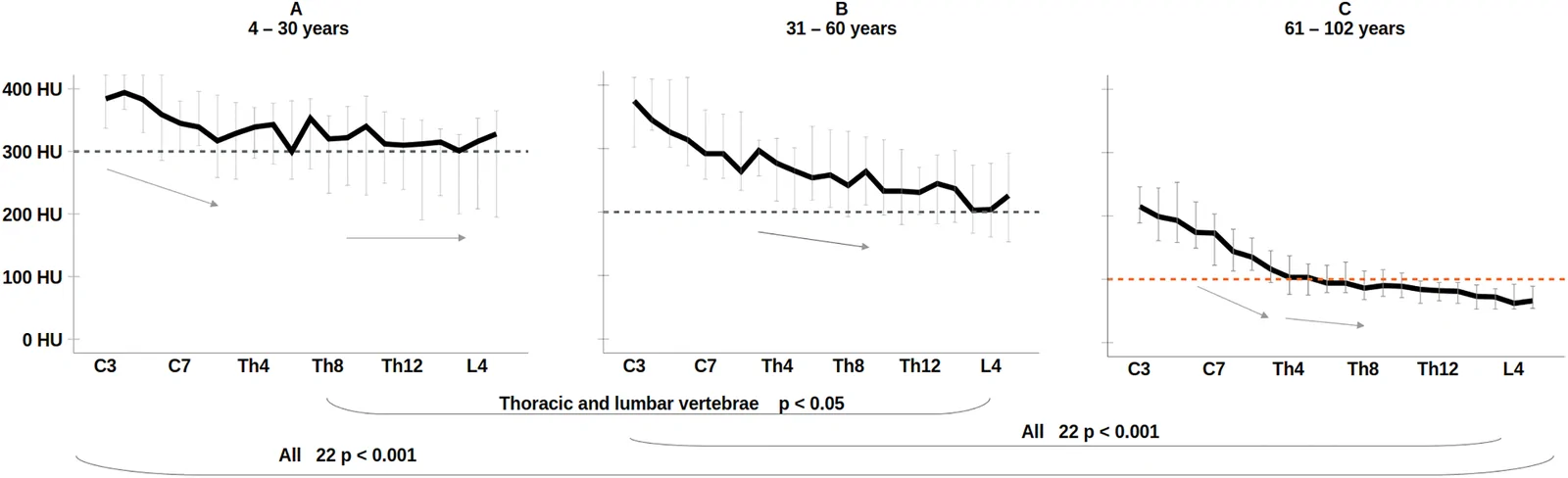
Bone density profiles (HU) from the cranial to the caudal aspect (C3-L5) in age. (A) In young individuals, we found an overall median profile ≥ 300 HU. In the upper spine, bone density reduced by a maximum of 94 HU, but in the lower spine it remained at a median level > 300 HU (n = 15: 6f, 9m). (B) Between the ages of 31 and 60 years, bone density was still high in the upper spine, and reduced consistently as one proceeded to the caudal aspect (n = 22: 6f, 16m). (C) In persons older than 60 years (n = 34: 22f, 12m; 79% OP), we found a reduced level of bone density, which was lower in all 22 vertebrae (C3-L5) than it was in younger individuals (p < 0.001). The reference value of 100 HU for osteoporosis was first undercut at Th6. Medians with their 95% confidence intervals. n – sample size, f – female, m – male, OP – osteoporosis.

### 3.2 Sex-related HU levels and profiles in the age groups

If cancellous bone density is considered independently of age, men show higher values in HU (p = 0.040) and BMD (p = 0.036) than women. However, age does influence the reduction of bone density. Comparing the HU levels between the three age groups, women show different levels exclusively between the last two age groups (300 vs. 108 HU, p < 0.001; medians from S2 Table), but not between both first age groups (329 vs. 300 HU, p = 0.699). The aggregated female HU values here were comparable high. In contrast, the male cohort aged > 19 years show significant falling levels (348 vs. 260 vs. 139 HU, p = 0.025 and p < 0.001).

HU profiles from C3-L5 (Fig 3) of older-aged women and men show that HU values reduce significantly for both sexes likewise, but they are not necessarily higher in men than in women in younger age, and women in young age do not routinely experience a loss of cancellous bone. Young women (4–30 years) revealed stable HU levels through the entire spine: C3 = 330 HU; L5 = 326 HU (Fig 3A), whereas young men from the age of 19 years showed a drop in median HU values from the cranial to the caudal aspect: C3 = 371 HU (292, 463); L5 = 328 HU (186, 358); p < 0.001. They had (with 348 HU) higher median values than young women (329 HU) (S2 Table, framed values). In the middle age group (31–60 years), both sexes revealed falling bone density profiles (p < 0.001 both) (Fig 3B); women had higher median values (300 HU) than men (260 HU). One male spine (51 years) was osteoporotic. Finally, persons older than 60 years revealed falling profiles with a consistent distance between sexes (Fig 3C): both groups achieved the highest median HU values in the cervical spine (men: C5 = 253 HU (176, 289) vs. women: C3 = 217 HU (126, 269)), with a continuous loss of cancellous bone to the lumbar spine (p < 0.001 both). Notably, women had values below the critical osteoporosis threshold of 100 HU even in the mid-thoracic spine: Th4 = 94.5 HU (73.3, 142), whereas men fell below this threshold value only at the thoracolumbar junction: Th11 = 97.0 HU (78.0, 156). 19/22 female spines and 8/12 male spines were osteoporotic.

**Fig 3:**
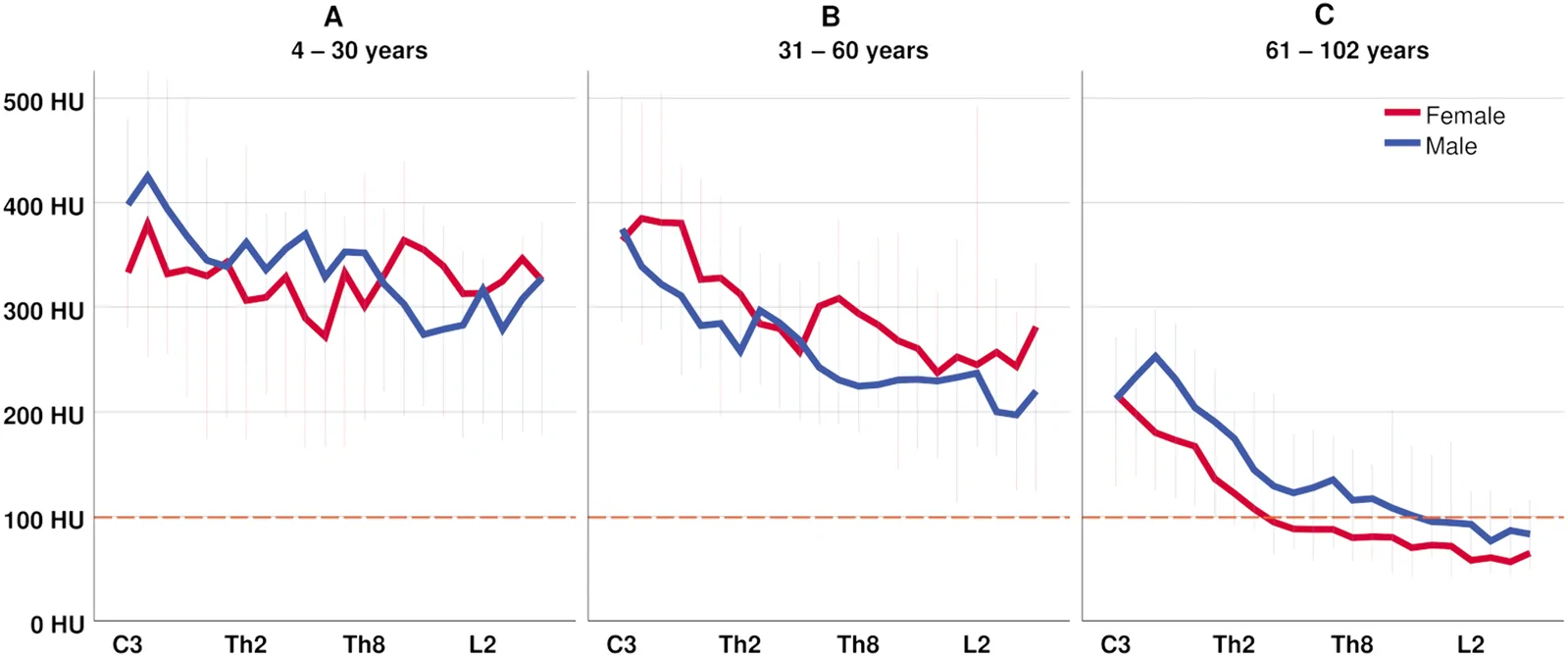
Gender-specific bone density profiles (HU) along the spine for three age groups. The male cohort (blue) did not show basically higher bone densities, but male profiles showed reducing HU levels between all age groups and falling trends. In women (red), bone density level was reduced along the spine only after the age of 30 years. (A) Women from 4 to 30 years (n = 6) revealed a steady density state, whereas young men from the age of 19 years (n = 9) showed a significant drop in HU values from the cranial to the caudal aspect (p < 0.001). (B) In persons aged 31–60 years, bone density fell along the spine (p < 0.001 both). Women (n = 6) showed higher bone densities (HU) than men (n = 16), with one osteoporotic male spine. (C) In the age group of those older than 60 years, both sexes have reduced cancellous bone densities from the cranial to the caudal aspect (p < 0.001 both). All vertebrae in men (n = 12) showed higher HU medians than in women (n = 22). Women in this age group (86% with OP) fell below the critical osteoporosis threshold of 100 HU (orange reference line) from the mid-thoracic spine (Th4), whereas men (67% with OP) achieved this value only at the thoracolumbar junction (Th11). Medians with their 95% confidence intervals.

### 3.3 Age-related loss of cancellous bone slowed down 8 years later for women than for men

Our three merged samples allowed for a depiction of age-related HU courses on a single value level from 4–102 years. In both sexes, every vertebra (C3-L5) showed a general reduction of HU with advancing age. However, the age-related courses of HU values represented by smooth data regression fits differed between the two cohorts, as shown in (Fig 4) exemplarily for Th9: data from females aged 4–102 years showed a parabolic curve from young to middle age and then a reduction, whereas male data (19–91 years) showed a reduction from the begin. The different courses (parabolic vs. linear) were seen in all investigated vertebrae (with exceptions of female L4 and L5). Seen courses are in line with the above reported significant reduction of age-aggregated HU levels only between middle aged and older women, but not between young and middle aged ones, vs. significant reductions of male HU levels between all age groups. The peak of the estimated regression fit around 30 years (Fig 4, blue dashed line) reveals slightly higher (< 50) HU in female spines than in male ones (cf. S2 Table). Both peaks around 30 years correspond to the calculated maximum median of 348 HU in the third decade of age, independently of sex (S2 Table).

**Fig 4:**
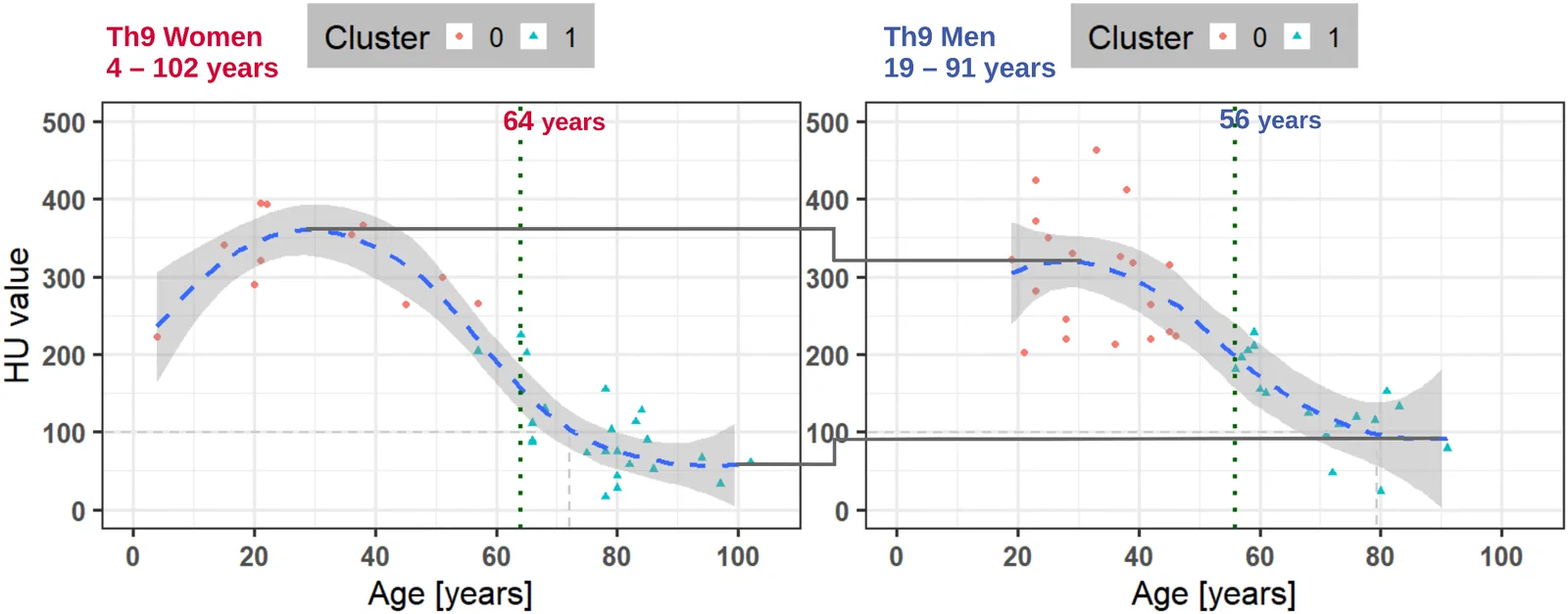
Reduction of cancellous bone density (HU) with age in Th9. The cluster analysis revealed young persons with high HU values (Cluster 0, red dots) and older persons with lower values (Cluster 1, green triangles). The cut-off age for Th9 was 64 years in women and 56 years in men (vertical dotted line). Two cohorts (4–102 years aged women vs. 19–91 years aged men) are shown. The female cohort showed a higher peak of the regression curve (blue dashed line with grey confidence bands) than the male cohort, both at the age of around 30 years, and a lower level in old age. They underwent the OP reference value of 100 HU earlier (70+) than men (almost 80 years). OP – osteoporosis.

For a more detailed characterization of age-related changes in cancellous bone density (HU) in women and men, we performed a cluster and ROC curve analysis for each vertebra from C3 to L5 in order to determine the age from which the loss of cancellous bone density changed and slowed down (Fig 4). In women we registered a deceleration of cancellous bone loss from the age of median 64 years (Q_1_ = 57, Q_3_ = 65). The corresponding age for men was markedly lower (p < 0.001), namely 56 years (45, 57). Especially in the lower spine, the calculated ages determined for each vertebra were consistent from Th12 to L5 (between 64 and 66 years in women; between 56 and 59 years in men). Even in the upper male spine (C3 to Th4, except for C6) we noted consistent age values, but not so in the mid-thoracic region.

### 3.4 Comparable impact of age on cancellous bone density (HU) and BMD (mg/cm^3^)

In general, HU values correlate with BMD and BV/TV data as well, which reports comparability of HU measurements with data of the two different imaging procedures, all describing bone density. HU correlates with BMD (each as mean of L1-L3): r = 0.86, 95% CI: 0.72–0.94, p < 0.001, and with BV/TV (each as mean of Th12, L1, L2): r = 0.51, 95% CI: 0.17–0.75, p = 0.006.

The influence of age (years) on HU and BMD (each with means L1-L3) is similar. The standardized regression coefficients which allow for comparison of different procedures show a slightly more rapid reduction of HU bone density with age (b^0,1^_HU_ = −0.814 and b^0,1^_BMD_ = −0.706, p < 0.001 both). The reduction of HU values with age was seen in both sexes ((b _women_ = −3.679 HU/year and b _men_ = −3.382; p < 0.001 both), indicating a more rapid loss in women. A critical threshold of 100 HU, which is regarded as an indicator of OP, was first undercut during the 8^th^ decade, with women reaching values < 100 HU earlier than men. BMD also reduced with age in both sexes (b _women_ = −1.921 mg/cm^3^/year and b _men_ = − 1.762, p < 0.001 both), with women achieving values below the OP threshold of 80 mg/cm^3^ before men, too.

### 3.5 Micro-CT showed a notable loss of cancellous bone earlier than computed tomography

To focus on bone density in vertebrae of potential fracture risk, HU measurements of Th12-L2 were analyzed related to three age groups and compared to BV/TV values as micro-CT-based measurements of volumetric bone density (Fig 5). A notable aspect, comparing HU and BV/TV of frequently fractured thoracolumbar junction, was the significant drop in HU values in those older than 60 years (vs. 31–60 years, p < 0.001 for each vertebra, cf. Fig 2), while BV/TV determined on micro-CT showed a marked reduction of values at the early age of 31 years and beyond (vs. 4–30 years, p ≤ 0.021 each).

**Fig 5:**
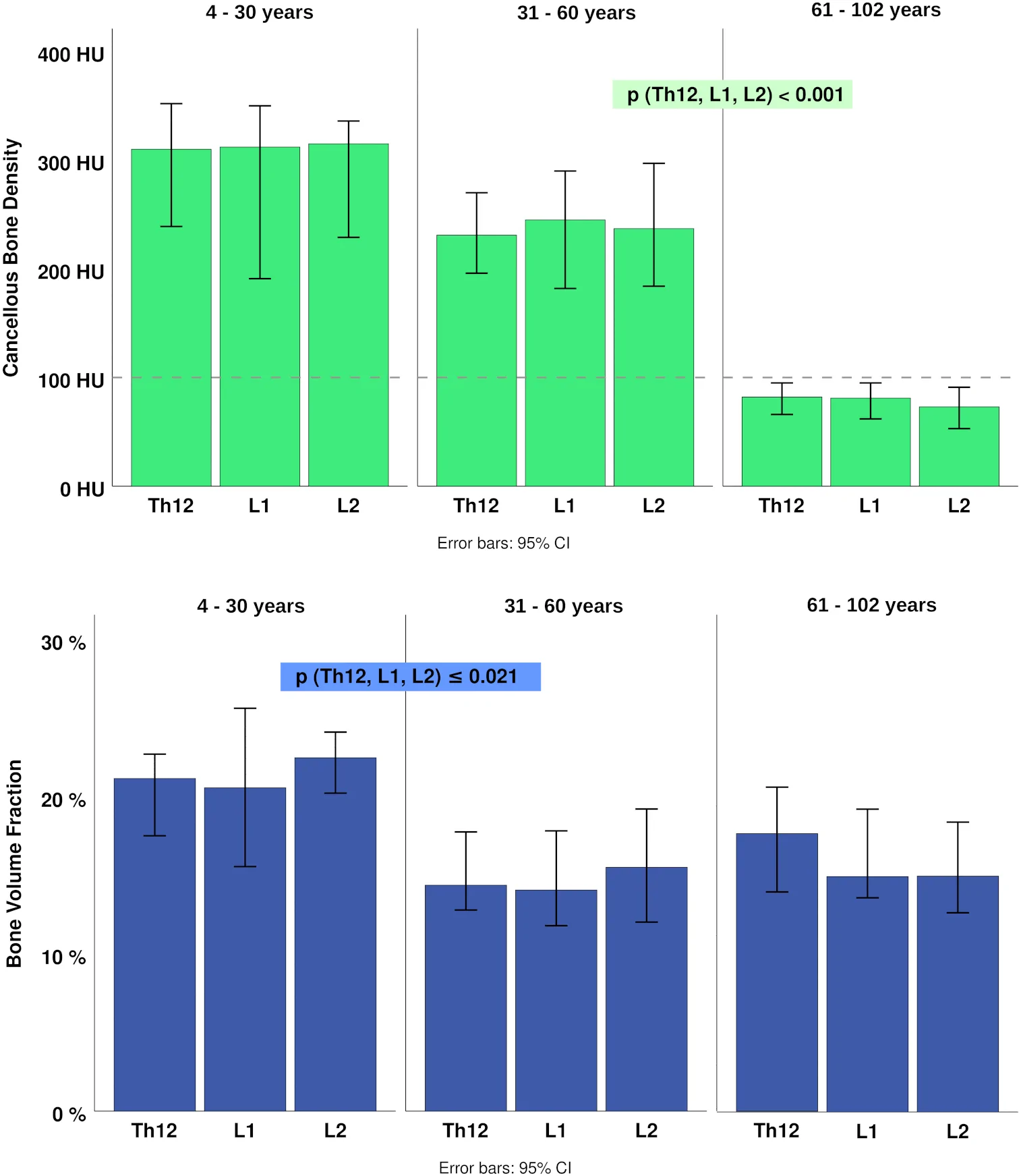
Thoracolumbar density in age groups. (A) Comparison of Hounsfield units (HU) determined on CT between the three age groups (4–30 years, n = 15; 31–60 years, n = 22; 61–102 years, n = 34) at the thoracolumbar junction (Th12, L1, L2). A significant drop in HU in those older than 60 years in all three investigated vertebrae was seen (p < 0.001 for each), with bone density below the 100 HU reference value for the diagnosis of osteoporosis. (B) Comparison of bone volume fraction (BV/TV, %) determined on micro-CT between the three age groups (n = 5; n = 18; n = 27). A notable finding was the significant drop in BV/TV even in the group above 30 years of age (p ≤ 0.021 for Th12, L1, L2). Medians with their 95% confidence intervals.

## 4 Discussion

The present investigation confirms and extends existing knowledge about bone loss with age, covering 4–102 years, and analyzing 71 complete C3-L5 HU profiles. We corroborate our HU results on the basis of established BMD and BV/TV parameters. Further, we describe sex-related changes of HU with age.

Our data show that bone density measurements in HU are more clearly correlated with BMD values (r = 0.86) than with BV/TV measurements (r = 0.51). The correlation of HU values with data obtained on micro-CT and quantitative CT data underlines the physical and biological comparability of the measurements, as well as supports the suitability of HU values from standard CT investigations as a surrogate parameter of trabecular bone quality [12]. Recent studies confirm the correlation between HU, BMD and microarchitectural parameters. Andresen et al. (2025) [11] showed that HU-based measurements from routine CT could be reliably converted into quantitative BMD values, thus serving as a valid and widely available tool for the diagnosis of osteoporosis, and especially permitting the assessment of fracture risk in the spine. Nagata et al. [19] considered mean DEXA Z-scores (for hip) and mean HU on CT (L1 – L5) in Propensity-matched cohorts of young patients vs. controls from 4–17 years (n = 21 each). They found a moderate correlation (r = 0.65) and stated HU as a reasonable proxy for BMD in the pediatric population. Wang et al. [20] and Lee et al. [21] confirm the significant association between HU and BMD in large patient populations and emphasize the fact that age and sex are significant factors influencing bone density. According to a systematic overview performed by Pinto et al. [22], HU values from CT investigations revealed a strong correlation with BMD values obtained from DXA studies and serve as a suitable diagnostic instrument for osteoporosis.

We demonstrate that the reduction of trabecular density is markedly determined by age. HU as well as BMD values reduce significantly with advancing age; regression analyses showed similar bone loss gradients in both procedures. The present investigation confirmed the known craniocaudal density gradients along the spine [8,15,17,23]. The highest HU values were consistently noted in the cervical spine, and the lowest values in the lumbar spine. This conforms to the varied trabecular microarchitecture and the regional differences in mechanical loads [17,24]. The loss of bone density increased with advancing age, especially at the thoracolumbar junction in the spine, which is the clinical zone of predilection for osteoporotic fractures.

Simion et al. [23] investigated bone density along the entire spine (cervical, thoracic and lumbar) in 200 probands aged 10–89 years. Differently from us, they worked on the basis of HU values registered on contrast-enhanced CT scans, calculated all of their trabecular bone density values (mg/cm^3^) with HU measurements, and used a sample unbalanced in sex (3:1) and an age cut at 50 years. The authors evaluated a total of 4,350 vertebrae and reported a significant reduction of mean bone density and a markedly lower bone density levels in all segments of the spine in persons older than 50 years compared to younger individuals. This age-related reduction concerned men as well as women and was especially noticeable after the age of 50 years. Both genders achieved the highest HU values in the CS, followed by declining values as one proceeded towards the LS. In this older age group, women fell below the critical osteoporosis value of 100 HU even in the mid-thoracic region (cf. Th4, Fig 3C), whereas men achieved this threshold value only from the thoracolumbar junction onward (cf. Th11). Our data show (in C3-L5 profiles and tables) a switch of sex-related HU levels: women aged 31–60 years had, on median, 40 HU higher values than men, and in the age group beyond 60, a 31 HU lower median than men. Simion et al. [23] depicted a switch of aggregated medians per decade during the 6^th^ decade of age. Their shown HU differences by sex didn’t also reach statistical significance though a threefold sample size. Simion et al. [23] performed their CT investigations with contrast medium, which may have influenced their HU data decreasing with ongoing age of patients. Contrasted CT’s were not performed in the present study.

However, we described differences between age-related HU courses of the two cohorts of 4–102 years old women vs. 19–91 years old men (Fig 3). While the latter one revealed a nearly steady reduction of HU values beginning from the age of 19 years, the first one showed a biphasic course from childhood onward: an increase in cancellous bone density during growth and early adulthood, with a maximum around the third decade of life, followed by a steep drop from middle age onward. Seen different HU courses of both cohorts with increasing age are in line with our results of the analysis of HU values aggregated in age groups, even if analyzing HU levels of both cohorts for exclusively age > 19. Further, different high peaks of both courses around an age of 30 years, and different ages when the HU reduction is slowing down match with estimated regression coefficients of sexes. But the attribution of seen differences to either age, sex and/or both can’t be specified by our data set. Especially, the small samples of young donors in sex subgroups do neither allow for a pair-match, nor for a more detailed age scaling to investigate HU in pediatrics. The measurement (209 HU) of the female case of age 4 might be as low by chance. An HU increase and difference in sex densities in young age remain descriptive here, but seem to match with findings of Desiree et al. [25], which analyzed aggregated values of L1 – L4 of 7,511 healthy Mexican children and adolescents aged 5–18 years and provided bone related percentiles for the pediatric Mexican population as references. They reported an increasing density of areal BMD values (g/cm^2^) and BMA(apparent)D (g/cm^3^) beginning around age 10 in both, girls and boys, till the age of 18 showing age-related smooth trends. This increase goes with our depiction of increasing HU in young females until 30 years, for each of C3 – L3. And, Desiree et al. [25] depict higher density for girls aged 18 than for boys (p50, Mexicans), as we do (estimated regression line, Germans). Interestingly, provided reference values (5–18 years) showing an increase in male aBMD and BMAD in adolescents from 10 to 18 may help to bridge our gap in male HU data before 19 years (all in conjunction with the reported correlation of BMD and HU values). Rauch and Schoenau [26] reported that trabecular BMD in vertebral bodies increases by about 40% between the ages of 2 and 20 years. Previous studies have shown that there are specific phases of life during which women achieve a level of bone density comparable to that of men – especially as young adults before menopause [27–29]. Gender-specific differences are seen primarily due to differences in bone geometry (men have larger bone volumes) and due to the markedly greater loss of bone density in women after menopause [28–30]. A large population-based DXA study (n = 46,381) in the US reported comparable density courses (smoothing splines) in age: an increase from the age of 8 in LS, with descriptively higher female densities till the age around 50, followed by a sex-related switch with female values declining below male densities [31]. A parabolic course with a peak around 35 years and a marked, hormone‑driven acceleration of female bone loss after menopause is also shown by Zeng et al. [32] analyzing data of 34,377 Chinese. Different sex courses of BMD from the age of 20 (a linear decline in men vs. pre-postmenopausal changes in woman) are shown by Warming et al. [33] for Danish people as well. These patterns, and also the adjusted results for lifestyle-related risk factors such as smoking, alcohol, low BMI and lack of exercise [34–36], are in line with our observation that women reach critical osteoporotic HU thresholds earlier than men and that the deceleration of HU loss occurs later in women (median 64 vs. 56 years). This could be a result of delayed metabolic adaptation after menopause or could have been a cohort effect in the investigated population.

In addition to HU and BMD, we show micro-CT data. BV/TV fell significantly at an early time point, i.e., between the first two trimesters of life, whereas the HU reduction became evident only between the last two trimesters. Galassi et al. [37] showed in their investigation of trabecular vertebral architecture in an archaeological human population that microscopic structural parameters are highly sensitive to the temporal dynamics of bone degeneration. In fact, the present investigation clearly brings forward the time point of microarchitectural changes. A combination of the two procedures may help to register early stages of osteoporosis more clearly and identify structural changes prior to the occurrence of fractures. Therefore, this “early warning function” of microarchitectural parameters should not remain unused. Rather, it should be employed early as an integrative measure and a supplementary imaging procedure for differentiated risk stratification.

### 4.1 Limitations

*This investigation has certain limitations. Obviously, a longitudinal in-vivo study is not feasible here, so we resorted to a cross-sectional investigation based on primarily post-mortem tissue, which, admittedly, cannot reflect dynamic bone loss processes. Our pragmatic approach of merging available data of different sources leads to partially missing medical histories and features for sample matching, and to quite moderate sample sizes per age and sex subgroup in young age given heterogeneity of the measurements. The small pediatric and adolescent subgroup (n* = *2, age < 19 years) may be sampling biased, and the findings here remain descriptively so far. The specificity of* HU *measurement in pediatric and adolescent population (i.e., in special* ROI *setting) should be accommodated for. Missing male data < 19 years do neither allow for a fair comparison with data of females (4–30 years) in the youngest age group nor for sex-related interpretation of the results.* HU *changes in > 60 years aged persons are to be interpreted with respect to age and* OP *(79%). Ethanol fixation and preservation may slightly alter tissue density measurements. Furthermore, the combination of clinical* CT, QCT*, and micro‑*CT *techniques, although carefully standardized, introduces minor systematic differences in spatial resolution and calibration. Finally,* BV/TV *analyses were restricted to thoracolumbar levels, whereas* HU *data covered the entire spine, limiting regional comparability.*

## 5 Conclusions

Cancellous bone density measured in Hounsfield units along the spine is correlated with established parameters such as BMD and BV/TV. Age does significantly impact the reduction of HU density level. HU profiles along the spine change with age in interaction with sex. Density loss of bone with age is also significant in BMD and BV/TV, with an earlier reduction detected in the microarchitecture. HU measurements serve as a practical and retrospectively applicable approach for the assessment of trabecular bone quality and the potential risk of osteoporosis in routine diagnostic studies.

## Supporting information

S1 Table Bone density values in Hounsfield units in vertebral bodies extracted from the cervical (C3-7), thoracic (Th1-12), and lumbar (L1-5) spine for both sexes (female/male). (PDF)

S2 Table Bone density values in HU for 10-year periods and age groups (4–30, 31–60, 61–102 years) of sexes (female/male), and for cervical (C3-7), thoracic (Th1-12), and lumbar (L1-5) spine regions. Features are framed.
(PDF)

## Abbreviations

ACR: American College of Radiology
BMD: bone mineral density (mg/cm^3^)
BV/TV: Bone Volume over Tissue Volume
cm: centimeter
CS: cervical spine
CT: computed tomography
C3-7: cervical vertebra 3-7
DXA: dual-energy X-ray absorptiometry
Fig: Figure
g/cm^3^: gram/cubic centimeter
GE: General Electric
HU: Hounsfield units
IBM: International Business Machines Corporation
kV: kilovolt
L1-5: lumbar vertebra 1-5
LS: lumbar spine
mg/cm^3^: milligram/cubic centimeter
micro-CT: micro-computed tomography
mio.: millions
ml: milliliter
mm: millimeter
N: number
OP: osteoporosis
PMMA: polymethyl methacrylate
QCT: quantitative computed tomography
ROC: receiver operating characteristic
ROI: region of interest
Th1-12: thoracic vertebra 1-12
TS: thoracic spine
VF: vertebral fractures
VOI: volume of interest
μm: micrometer
μA: microampere
◦C: degrees Celsius
Δ: delta, difference
