# Supplementary material for: Investigation of age- and sex-related changes in cancellous bone density in the human spine using CT
Source: PLoS One. 2026 Sep 28;21(9):e0358344. doi: 10.1371/journal.pone.0358344 (PMC13618916; doi:10.1371/journal.pone.0358344)
Supplement: Supplementary file 1 [file pone.0358344.s001.pdf]

S1 Table. Bone density values in Hounsfield units in vertebral bodies extracted from the cervical (C3-7), thoracic (Th1-12), and lumbar (L1-5) spine for both sexes (female/male)

	HU values of 71 spines per vertebra				
**Location**	**N**	**Mean**	**Median**	**Q_1**	**Q_3**
C3	69	300	287	213	382
C4	69	300	281	213	378
C5	71	287	289	192	360
C6	71	267	263	174	352
C7	71	248	241	172	345
**Cervical**	**71**	**279**	**266**	**184**	**354**
f / m	34 / 37	249 / 305	231 / 319	167 / 237	333 / 367
Th1	71	239	234	144	337
Th2	71	225	210	135	309
Th3	71	219	219	116	307
Th4	71	211	203	103	315
Th5	70	206	197	105	288
Th6	70	202	191	98	281
Th7	70	204	187	96	313
Th8	70	193	185	88	279
Th9	70	197	202	95	293
Th10	70	195	185	91	287
Th11	70	186	167	86	278
Th12	70	182	177	84	264
**Thoracic**	**71**	**204**	**198**	**104**	**300**
f / m	34 / 37	174 / 230	139 / 228	82 / 158	265 / 323
L1	71	180	175	83	273
L2	71	177	165	74	278
L3	71	166	157	72	255
L4	69	162	137	62	250
L5	69	169	149	66	278
**Lumbar**	**71**	**172**	**159**	**75**	**259**
f / m	34 / 37	149 / 194	92 / 193	56 / 119	249 / 276
**Total C3-L5**	**71**	**216**	**209**	**115**	**307**
f / m	34 / 37	186 / 239	161 / 239	96 / 169	265 / 324
