# Supplementary material for: Investigation of age- and sex-related changes in cancellous bone density in the human spine using CT
Source: PLoS One. 2026 Sep 28;21(9):e0358344. doi: 10.1371/journal.pone.0358344 (PMC13618916; doi:10.1371/journal.pone.0358344)
Supplement: Supplementary file 2 [file pone.0358344.s002.pdf]

S2 Table. Bone density values in HU for 10-year periods and age groups (4-30, 31-60, 61-102 years) of sexes (female/male), and for cervical (C3-7), thoracic (Th1-12), and lumbar (L1-5) spine regions

	HU values of 71 spines age-related				
	**N**	**Mean**	**Median**	**Q_1**	**Q_3**
**Age in decades**					
0-10	1	209	209		
11-20	3	344	336	322	
21-30	11	328	348	278	391
31-40	7	351	345	331	371
41-50	6	263	255	206	324
51-60	9	237	246	199	260
61-70	8	178	187	130	214
71-80	15	104	108	79	126
81-90	7	129	126	97	167
91-102	4	79	84	57	97
**Age groups**					
**4-30 years**	**15**	**324**	**336**	**278**	**374**
f / m	6 / 9	320 / 326	**329 / 348**	272 / 273	375 / 382
C		378	363	330	440
Th		318	320	268	362
L		281	308	202	335
**31-60 years**	**22**	**282**	**260**	**229**	**345**
f / m	6 / 16	295 / 277	**300 / 260**	239 / 209	350 / 331
C		332	330	266	401
Th		259	242	204	325
L		232	210	165	290
**61-102 years**	**34**	**124**	**109**	**91**	**166**
f / m	22 / 12	119 / 135	**108 / 139**	84 / 98	159 / 172
C		196	185	137	246
Th		112	103	76	143
L		83	68	52	92

Features are framed.
